# Protein-based prognostic signature for predicting the survival and immunotherapeutic efficiency of endometrial carcinoma

**DOI:** 10.1186/s12885-022-09402-w

**Published:** 2022-03-25

**Authors:** Jinzhi Lai, Tianwen Xu, Hainan Yang

**Affiliations:** 1 Department of Oncology, The Second Affiliated Hospital of Fujian Medical University, Quanzhou, 362000 Fujian China; 2 Department of Ultrasound, First Affiliated Hospital of Xiamen University, Xiamen, 361000 Fujian China

**Keywords:** Endometrial carcinoma, Proteomics, Prognostic signature, Tumor-infiltrating immune cells, Treatment

## Abstract

**Background:**

Endometrial cancer (EC) is the most frequent malignancy of the female genital tract worldwide. Our study aimed to construct an effective protein prognostic signature to predict prognosis and immunotherapy responsiveness in patients with endometrial carcinoma.

**Methods:**

Protein expression data, RNA expression profile data and mutation data were obtained from The Cancer Proteome Atlas (TCPA) and The Cancer Genome Atlas (TCGA). Prognosis-related proteins in EC patients were screened by univariate Cox regression analysis. Least absolute shrinkage and selection operator (LASSO) analysis and multivariate Cox regression analysis were performed to establish the protein-based prognostic signature. The CIBERSORT algorithm was used to quantify the proportions of immune cells in a mixed cell population. The Immune Cell Abundance Identifier (ImmuCellAI) and The Cancer Immunome Atlas (TCIA) web tools were used to predict the response to immunochemotherapy. The pRRophetic algorithm was used to estimate the sensitivity of chemotherapeutic and targeted agents.

**Results:**

We constructed a prognostic signature based on 9 prognostic proteins, which could divide patients into high-risk and low-risk groups with distinct prognoses. A novel prognostic nomogram was established based on the prognostic signature and clinicopathological parameters to predict 1, 3 and 5-year overall survival for EC patients. The results obtained with Clinical Proteomic Tumor Analysis Consortium (CPTAC), Human Protein Atlas (HPA) and immunohistochemical (IHC) staining data from EC samples in our hospital supported the predictive ability of these proteins in EC tumors. Next, the CIBERSORT algorithm was used to estimate the proportions of 22 immune cell types. The proportions of CD8 T cells, T follicular helper cells and regulatory T cells were higher in the low-risk group. Moreover, we found that the prognostic signature was positively associated with high tumor mutation burden (TMB) and high microsatellite instability (MSI-H) status in EC patients. Finally, ImmuCellAI and TCIA analyses showed that patients in the low-risk group were more inclined to respond to immunotherapy than patients in the high-risk group. In addition, drug sensitivity analysis indicated that our signature had potential predictive value for chemotherapeutics and targeted therapy.

**Conclusion:**

Our study constructed a novel prognostic protein signature with robust predictive ability for survival and efficiency in predicting the response to immunotherapy, chemotherapy and targeted therapy. This protein signature represents a promising predictor of prognosis and response to cancer treatment in EC patients.

**Supplementary Information:**

The online version contains supplementary material available at 10.1186/s12885-022-09402-w.

## Background

Endometrial cancer (EC) is the most common gynecologic malignancy of the female genital tract in the world [1]. Although the incidence of EC is increasing, outcomes for patients are favorable because of the early symptoms of irregular vaginal bleeding, which trigger patients to seek care when the cancer is at an early stage [2]. However, the mortality rate for EC has increased more quickly than the incidence rate, with an estimated 76,000 deaths among women each year worldwide [3]. This increased disease mortality is associated with advanced stage, aggressive histology and metastasis [4]. It is difficult to reliably identify the patients with EC who are at the highest risk of recurrence and apply effective therapeutics. Despite advances in multidisciplinary and multi-institutional collaboration, progress and success with the development of prognostic markers remain limited. To date, there are no reliable biomarkers or predictive models to precisely predict the survival of EC patients [5]. To further improve the outcomes of EC patients overall, predictive and prognostic signatures to identify high-risk patients are critical.

EC consists of a heterogeneous group of tumor cells and proteins with distinct characteristics. Anatomical classification offers limited information for evaluating patient outcomes, and the response to treatment is difficult to predict by this system. Recent studies have demonstrated that proteins play critical roles in the pathogenesis and progression of EC [6]. With the development of high-throughput technologies, mass spectrometry-based proteomics allows the reclassification of cancer and may influence prognosis and guide clinical decision-making [7]. At present, proteomic and molecularly guided management of EC lags behind most other cancers [8]. Recently, protein-based prognostic signatures according to public databases have attracted wide attention and revealed huge potential in prognosis prediction for cancer patients [9–12]. Considering the importance of proteomics and the immune microenvironment in the carcinogenesis and progression of EC, the identification of a protein prognostic signature is necessary to provide novel insights into the biological behaviors of EC and benefit clinical treatments.

The use of newly developed proteomics technologies and bioinformatics approaches to investigate EC has provided unprecedented insight into cancer biology and treatment efficacy [13]. The Cancer Proteome Atlas (TCPA) database is an open-access bioinformatics data repository based on mass spectrometry that can quantitatively evaluate vast amounts of protein markers in thousands of samples in a fast, sensitive and cost-effective high-throughput way. Screening proteins of potential prognostic value is a key step for predicting the prognosis of patients and identifying new therapeutic targets. The current challenge is to check whether specific molecular characteristics can be matched for patient prognosis and therapeutics [14]. In this study, we established a protein-based prognostic signature to predict the individual prognosis of EC. To validate the prognostic signature, we investigated its efficiency and accuracy in the training and testing sets. The results obtained with Clinical Proteomic Tumor Analysis Consortium (CPTAC), Human Protein Atlas (HPA) and immunohistochemical (IHC) staining data from EC samples in our hospital supported the predictive ability of these proteins in EC tumors. In addition, immune cell infiltration and the tumor mutation burden (TMB) associated with this signature were explored. Our study demonstrated and proved that the protein-based prognostic signature can be applied in the clinical prognosis of EC patients. Importantly, our study provided a new approach for predicting the response to treatment, including immunotherapy, chemotherapy and targeted therapy, in EC patients.

## Methods

### Data collection

The protein expression data of EC were retrieved from TCPA (https://www.tcpaportal.org/tcpa/). RNA-seq expression profile data (HTSeq-FPKM) were downloaded from the TCGA data portal (https://portal.gdc.cancer.gov/cart). The corresponding clinical data, including age, survival time, tumor grade, clinical stage and microsatellite instability (MSI) status, were downloaded from the TCGA portal. Patients were randomly divided into a training set and a testing set at a ratio of 5:5 using the “caret” package. The distributions of clinical stage, follow-up time, age, and death rate were similar between the two datasets (Table S1). The training set was used to identify the prognostic proteins and establish the protein-based prognostic signature, and the testing set was used to validate its prognostic capability.

### Construction of the protein prognostic signature

To explore the possible proteins in relation to prognosis for EC patients, univariate Cox proportional hazard regression analysis was used to identify the relationship between prognostic proteins and OS in the training set, and *p* < 0.05 was considered to be statistically significant. After that, using the “glmnet” package [15, 16], prognostic proteins were evaluated by the least absolute shrinkage and selection operator (LASSO) to minimize overfitting and identify the most significant prognosis-related proteins. To choose the penalty parameter λ, tenfold cross-validation was conducted on the training set. The optimal penalty parameter was defined as the value within one standard deviation of the minimum cross-validated partial likelihood deviance to obtain the best model. A subset of proteins was obtained by shrinking the regression coefficient using a penalty proportional to their size. The proteins with nonzero regression coefficients were chosen for subsequent multivariate Cox regression analyses. Next, the regression coefficient obtained by multivariate Cox regression analysis was multiplied by the expression level of each protein to construct the protein-based prognostic signature. The following formula based on the combination of regression coefficient and protein expression level was used to calculate the risk score: Risk score = expression_*protein1*_ × β_*protein1*_ + expression_*protein2*_ × β_*protein2*_ + ⋯ + expression_*protein x*_ × β _*protein x*_, where β represents the coefficient index and *x* is the number of proteins. The risk score was calculated based on this model for each patient in the training set and testing set. Patients were divided into high-risk or low-risk groups according to the median value of risk scores.

### Performance assessment

The efficiency and sensitivity of survival prediction based on the risk score was verified by receiver operating characteristic (ROC) curve analysis. We calculated the area under the curve (AUC) at 1, 3 and 5 years using the “survival ROC” package and evaluated the significance of the survival difference between the high-risk and low-risk groups. Kaplan–Meier survival curve analysis was used to analyze the overall survival (OS) between the high-risk and low-risk groups. The concordance index (c-index) was applied to assess the accuracy of this signature in the training, testing and total sets. We calculated the C-index through bootstrap resampling to estimate model accuracy using the “dplyr”, “rms”, “survival” and “pec” R packages.

### Identification of prognostic factors and predictive nomogram construction

To identify independent prognostic parameters and to validate the independent prognostic value of the prognostic signature and clinicopathological parameters, including age, survival time, tumor grade, clinical stage and MSI status, univariate and multivariate Cox regression analyses were performed in the training and testing groups. After testing for collinearity, we formulated a prognostic nomogram consisting of all independent prognostic factors and relevant clinical parameters based on multivariate Cox regression analysis. The predictive accuracy of the nomogram was tested by presenting the difference between actual survival and predicted survival using a calibration plot.

### Verification of prognostic protein expression

The UALCAN website ( http://ualcan.path.uab.edu/cgi-bin/ualcan-res.pl) is the Clinical Proteomic Tumor Analysis Consortium (CTPAC) database data mining platform that provides the proteins expression level of EC patients. Human Protein Atlas (HPA) provides the protein level of prognostic proteins in tumor and normal tissues. For IHC studies, 100 formalin-fixed paraffin-embedded (FFPE) tumor samples of EC patients were obtained from the pathology department of the Second Affiliated Hospital of Fujian Medical University from January 01, 2011 to December 01, 2021. The diagnosis and clinical stage of EC were determined according to the International Federation of Gynecology and Obstetrics (FIGO) guidelines. Antibodies against ER (SP1; Roche) and PR (1E2; Roche) were used in this study. The expression of ER-α and PR proteins was determined by scoring the percentages of positive rate (ranging from 0 to 100%) tumor cells. This study was approved by the Ethics Committee of The Second Affiliated Hospital of Fujian Medical University. The need for written informed consent was waived due to the retrospective nature of this study.

### Estimation of tumor-infiltrating immune cells

The CIBERSORT algorithm was used to quantify the proportions of immune cells in a mixed cell population [17]. The RNA-Seq (FPKM format) of EC samples was analyzed to obtain the abundance ratio matrix of 22 immune cells in each sample, including macrophages (M1 macrophages, M2 macrophages, and M0 macrophages), T cell types (T follicular helper [Tfh] cells, resting memory CD4 + T cells, activated memory CD4 + T cells, γδ T cells, CD8 + T cells, Tregs, and naïve CD4 + T cells), resting natural killer (NK) cells, activated NK cells, resting mast cells, activated mast cells, memory B cells, resting dendritic cells (DCs), activated DCs, naïve B cells, monocytes, plasma cells, neutrophils and eosinophils [18]. The CIBERSORT results of samples with *p* < 0.05 indicated that the inferred fractions of immune cell populations produced by CIBERSORT were accurate and were eligible for further analysis. The CIBERSORT output estimates were normalized, and immune cell type fractions were summed up to one.

### TMB analysis

The mutation data of EC patients were downloaded from the TCGA data portal. The data were stored in the form of Mutation Annotation Format and processed by VarScan software. The “maftools” package was used to analyze and summarize these mutation data [19]. For each EC patient, we calculated the tumor mutation burden (TMB) score as follows: (total mutation/total covered bases) × 10^6^ [20].

### Prediction of immunotherapy response

Immune Cell Abundance Identifier (ImmuCellAI) is a computational method published in 2020 to predict the response to immune checkpoint blockade based on the abundance of immune cells, particularly different T cell subsets [21]. The abundance of infiltrating immune cells was calculated by ImmuCellAI and used to develop the response prediction model. The immunotherapy response prediction model was developed using a support vector machine with the radial basis function kernel. The Cancer Immunome Atlas (TCIA) web tool provides the results of comprehensive immunogenomic analyses. Tumor immunogenicity was quantitatively scored from 0 to 10 and was named the immunophenoscore (IPS). The IPS could be applied to predict the response to immune checkpoint inhibitors [22].

### Assessment of the sensitivity of chemotherapy and molecular drugs

To estimate the risk score in predicting the response to chemotherapy and molecular drugs, the “pRRophetic” R package was applied to calculate the half-maximal inhibitory concentration (IC50) of samples between the low-risk and high-risk groups. According to clinical recommendations, chemotherapeutic and molecular drugs such as paclitaxel, docetaxel, cisplatin, doxorubicin, PI3K/Akt/mTOR inhibitors and VEGF inhibitors were selected as candidate drugs. The IC50 between the low-risk and high-risk groups was compared by the Wilcoxon signed-rank test.

### Statistical analysis

Statistical analyses were performed using R software (version 3.6.3). All tests were two sided, and a *p* value of less than 0.05 was considered statistically significant unless stated otherwise. The survival curves were compared using the Kaplan–Meier method, and the log-rank test was used to evaluate the statistical significance of the survival rates between different groups. The hazard ratio (HR) and 95% confidence interval were calculated to identify genes related to OS. The Mann–Whitney U test was used to compare the differences between two groups. The predictive accuracy of the risk signatures was determined by ROC curves.

## Results

### Construction of a proteomic signature from the training set

**Fig. 1:**
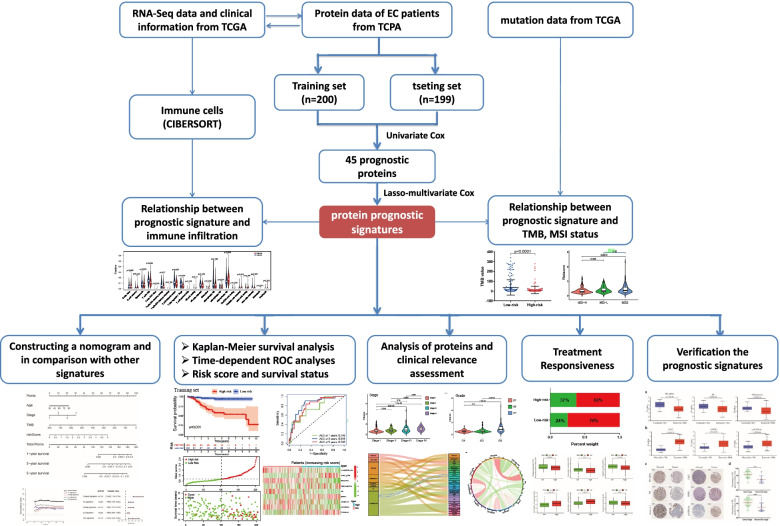
The workflow of this study

**Fig. 2:**
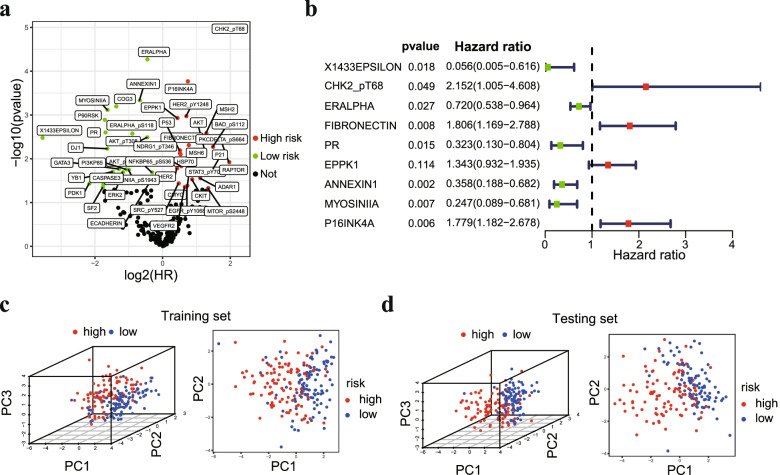
Construction of a prognostic signature from the training set. **a** Volcano plot of 45 proteins that were significantly associated with OS in EC. The red dots represent high-risk proteins, while the green dots indicate low-risk proteins. **b** Forest plot of the multivariate Cox regression analysis in the training set. **c** Principal component analysis based on the expression level of 9 prognostic proteins in the training set. **d** Principal component analysis based on the expression level of 9 prognostic proteins in the testing set

The workflow of our study is shown in Fig. 1. The whole dataset was divided into a training set (*n* = 200) and a testing set (*n* = 199). The training set was used to build the protein prognostic model, and the testing set and whole dataset were used to validate the prognostic model. To screen prognosis-related proteins, univariate Cox regression analysis was conducted to assess the prognostic characteristics of 223 proteins from the TCPA database. After screening, 45 proteins were identified to show a remarkable correlation with OS in the training set (*p* < 0.05) (Fig. 2a). Next, LASSO penalized Cox regression was applied to reduce the prognostic proteins, and tenfold cross-validation for penalty parameter selection is shown in Fig. S1a. Finally, 9 prognostic proteins with nonzero regression coefficients were included in the multivariate Cox regression analysis. We calculated the risk score of each patient based on the coefficient of each prognostic protein: Risk score = (-1.72179 × X1433EPSILON) + (0.02186 × Chk2-pT68) + (-0.1646 × ER alpha) + (0.08018 × Fibronectin) + (-0.14603 × PR) + (0.10249 × EPPK1) + (-0.05177 × Annexin 1) + (-0.71246 × Myosin IIA) + (0.15701 × p16INK4a 1). The forest plot of HR showed that X1433EPSILON, ER-alpha, PR, Annexin 1, and Myosin IIA were favorable prognostic proteins and that Chk2-pT68, Fibronectin, EPPK1, and p16INK4a were unfavorable prognostic proteins (Fig. 2b). Principal component analysis (PCA) showed that the prognostic signature could clearly categorize EC patients into two groups in both the training set (Fig. 2c) and testing set (Fig. 2d).

### Assessment the performance of the protein prognostic signature

**Fig. 3:**
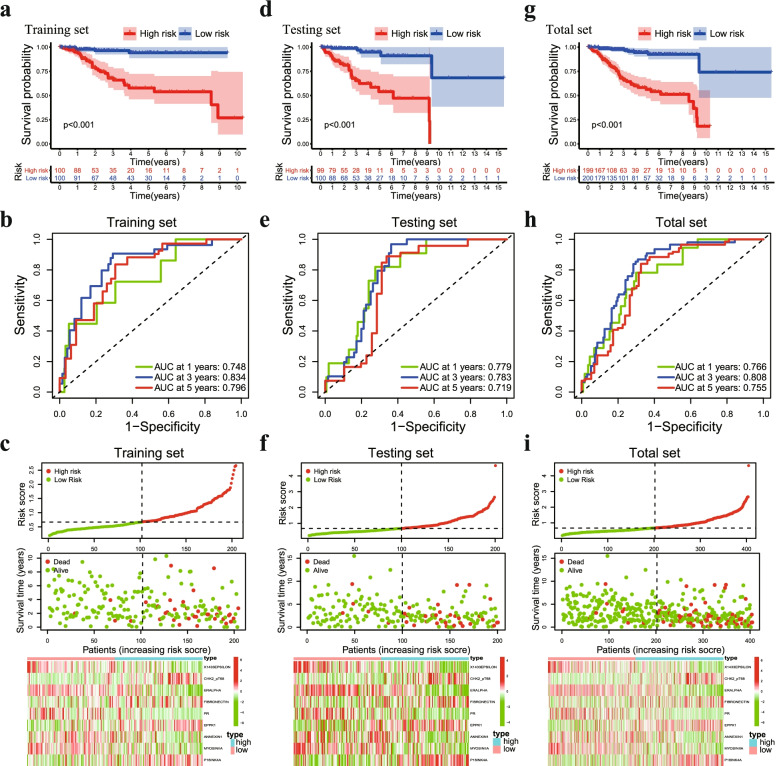
Assessment of the predictive power of the prognostic signature. **a** Kaplan–Meier analysis of OS in the high-risk and low-risk groups in the training set, **d** testing set and (**g**) total set. **b** Time-independent ROC analysis of risk scores for predicting OS in the training set, (**e**) testing set and (**h**) total set. **c** The distribution of risk scores, survival status and expression level of 9 prognostic proteins in the training set, (**f**) testing set and (**i**) total set

EC patients in the training set were divided into a high-risk group (*n* = 100) and a low-risk group (*n* = 100) according to the median risk score. Patients belonging to the low-risk group had a significantly better prognosis than patients in the high-risk group. Survival curve analysis showed that the low-risk group had longer OS than the high-risk group (Fig. 3a). The AUC for the immune-related risk signature was 0.784, 0.843 and 0.796 at 1, 3 and 5 years for OS (Fig. 3b). Patients in the training set appeared to have an increased mortality rate with an increase in risk scores according to the risk plot. In the high-risk group, Chk2-pT68, Fibronectin, EPPK1, and p16INK4a proteins were upregulated, while X1433EPSILON, ER-alpha, PR, Annexin 1, and Myosin IIA were downregulated (Fig. 3c).

Subsequently, we used the testing set (*n* = 199) and the total dataset (*n* = 399) to verify the accuracy of the signature. As expected, the Kaplan–Meier survival curves showed that OS was significantly longer in the low-risk group than in the low-risk group in the testing set (Fig. 3d). The ROC analysis showed that the 1-, 3- and 5-year AUC values were 0.779, 0.783, and 0.719, respectively (Fig. 3e). The distribution of the risk score, survival status, and expression of 9 proteins in the testing set were similar to those in the training set (Fig. 3f). For the total set, remarkable survival differences between the high-risk and low-risk groups were also verified (Fig. 3g), and the ROC analysis showed that the 1-, 3- and 5-year AUC values were 0.766, 0.808, and 0.755, respectively (Fig. 3h). The risk curve and protein profile heatmap showed similar characteristics to those of the training set and the testing set (Fig. 3i). In addition, the C-index of the prognostic signature was greater than 0.7 (Fig. S1b). These results indicated that the protein signature may be used as a prognostic biomarker for EC patients.

### Construction of a predictive nomogram based on the prognostic signature

**Fig. 4:**
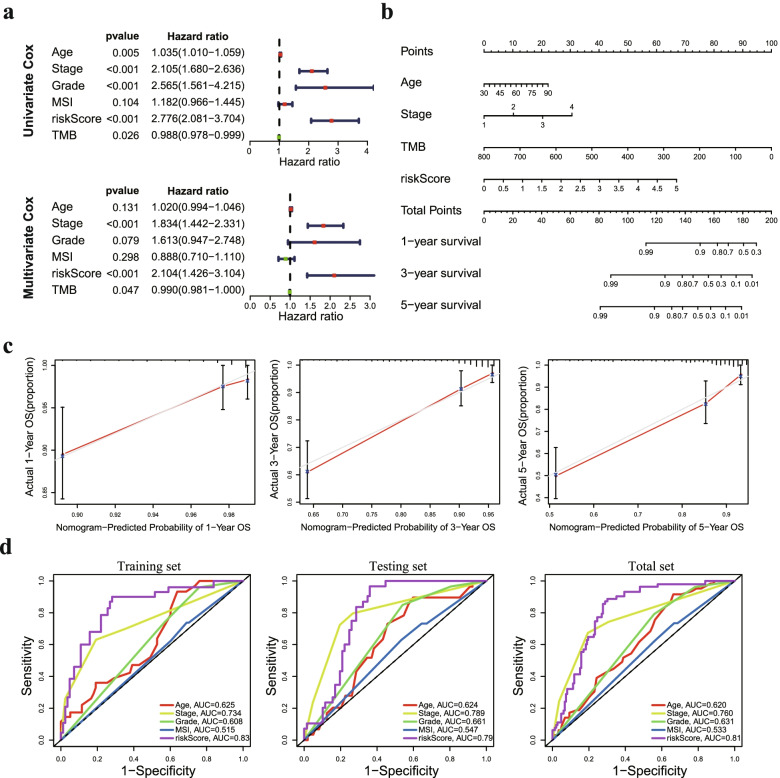
Identification of independent prognostic factors and establishment of the nomogram. **a** Univariate and multivariate Cox regression analyses to verify the prognostic values of various clinicopathological factors and risk scores. **b** A nomogram based on the prognostic signature consisting of risk score and clinical factors. **c** Calibration plot for evaluating the predictive accuracy of the nomogram at 1-, 3- and 5-year survival. **d** ROC analysis of the performance of the proteomic signature and clinicopathological factors

To assess the independent prognostic force of the prognostic signature, both univariable and multivariable Cox proportional hazard regression models were applied in the training and testing sets. The results from univariable and multivariable analysis demonstrated that the prognostic signature could be an independent predictor after other variables, including age, grade, stage and TMB status, were adjusted in the training set (Fig. 4a), testing set (Fig. S2a) and total set (Fig. S2b). To construct a suitable tool for clinical practice, we established a prognostic nomogram to predict 1-, 3- and 5-year OS using the protein prognostic signature and the independent clinicopathological factors we found above (Fig. 4b). The calibration curve demonstrated optimal predictive accuracy between predictive and actual values for the probabilities of 1-, 3- and 5-year survival, indicating the good ability to distinguish most survival outcomes at these time points (Fig. 4c). In particular, it is worth mentioning that the AUC of the protein signature was better than that of the existing clinicopathological characteristics, including age, grade, stage and MSI status (Fig. 4d). These results revealed that the predictive nomogram had good accuracy in predicting the survival of patients with EC.

Furthermore, we validated the performance differences between our signature and other previously reported gene signatures in EC. The AUC of present protein signature for 1, 3 and 5 year OS prediction was higher in comparison with Hu's signature [23], Huang's signature [24], Liu's signature [25] and Qin's signature [26] (Fig. S3a). Our model had the highest C-index value compared to other reported models (Fig. S3b). Notably, a forest plot showed that our protein signature was more powerful in predicting the prognosis of EC patients than other published prognostic signatures (Fig. S3c). These data indicated that the power of our prognostic signature seemed better than that of other previously reported studies.

### Clinical relevance assessment and construction of the protein coexpression network

**Fig. 5:**
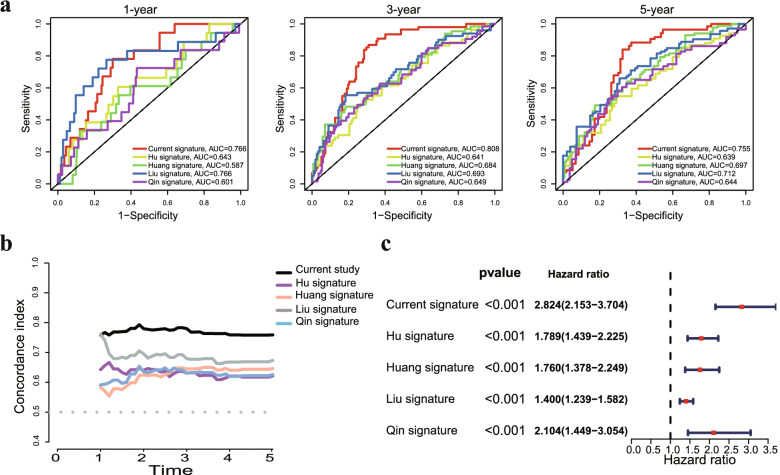
The relationship between 9 prognostic proteins, the risk score and clinical characteristics. **a** The scatter plot shows the correlation between the risk score and tumor stages. **b** The scatter plot shows the correlation between risk score and tumor grade. **c** The expression of EPPK1, p16INK4a, Annexin 1 and Myosin IIA was related to age in EC patients. **d** The expression of ER-alpha, Annexin 1, Chk2-pT68 and p16INK4a was significantly associated with cancer stage. **e** Sankey diagram of all proteins related to 9 proteins in the TCPA database (correlation coefficient > 0.4) (*p* < 0.001). (**F**) The corelationship of 9 proteins in the prognostic signature. * *p* < 0.05, ** *p* < 0.01, *** *p* < 0.001

We performed clinical correlation analysis to explore the correlation between 9 prognostic proteins, risk score and clinical characteristics in EC patients. We found that the risk score was significantly increased in patients with high-grade and high-stage tumors (Fig. 5a-b). The correlation analysis showed that the expression of EPPK1 and p16INK4a was increased in older patients (≥ 65 years), and Annexin 1 and Myosin IIA were more highly expressed in younger patients (< 65 years) (Fig. 5c). ER-alpha and Annexin 1 expression levels were higher in early-stage tumor tissues than in advanced-stage tumor tissues, while Chk2-pT68 and p16INK4a exhibited the opposite features (Fig. 5d). Other results of the clinical correlation analysis are shown in Fig. S4. Next, we carried out protein coexpression analysis for all 9 proteins, and 27 identified proteins with correlation coefficients > 0.4 and a *p* value < 0.001 were identified. All of these proteins are shown in the Sankey diagram (Fig. 5e). The correlation of the proteins included in the prognostic signature is displayed in Fig. 5f, in which ER-alpha and PR showed the strongest positive correlation, while ER-alpha and fibronectin showed the strongest negative correlation.

### Verification of the expression levels of prognostic proteins

**Fig. 6:**
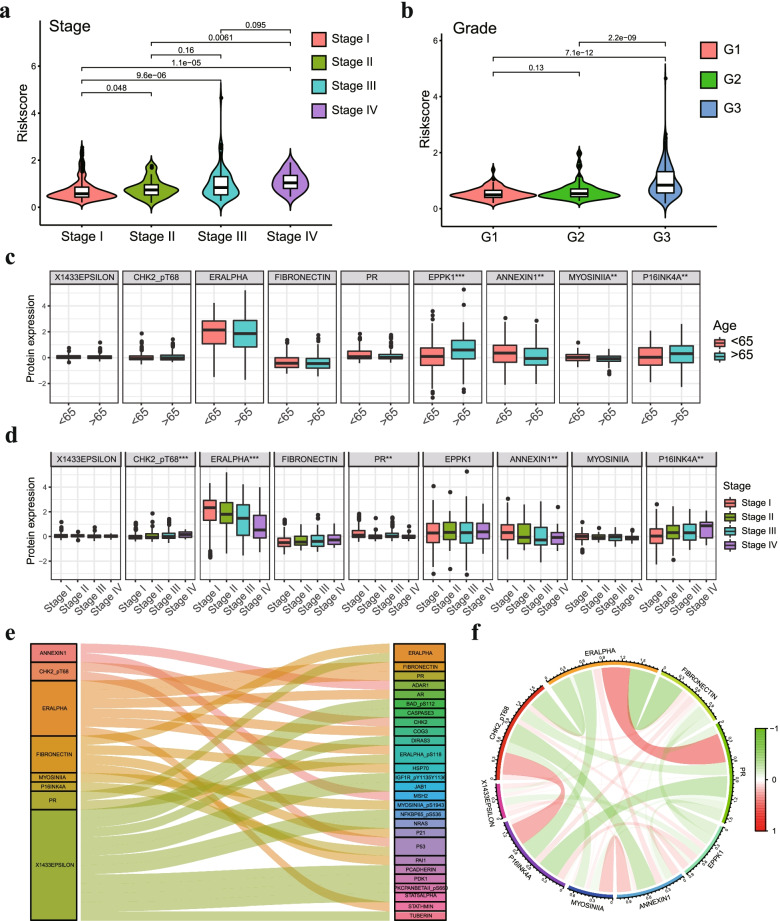
The relationship between 9 prognostic proteins, the risk score and clinical characteristics. **a** Protein level of prognostic proteins in EC tumor tissues and normal tissues. **b** Representative protein expression levels of ER-alpha, PR, Annexin 1, Chk2-pT68, EPPK1, p16INK4a and ASNS explored in the HPA database. **c** IHC staining data of ER-alpha and PR expression levels from 100 clinical samples in our hospital. * *p* < 0.05, ** *p* < 0.01, *** *p* < 0.001

We analyzed the expression of 9 prognostic proteins in EC and normal tissue from the CPTAC database. The ER-alpha, PR and Annexin 1 protein expression levels were increased in normal tissue compared with EC tissue, while the expression of Chk2-pT68, EPPK1 and p16INK4a was lower in normal tissue (Fig. 6a). In the HPA data, compared with normal tissues, Chk2-pT68, EPPK1 and p16INK4a were expressed at medium to high levels in tumor tissues, while the expression of ER-alpha, PR and Annexin 1 was significantly downregulated in EC tumors (Fig. 6b). In addition, immunohistochemistry (IHC) was applied to evaluate the expression levels of ER-alpha and PR in EC patients. IHC staining data from 100 clinical samples in our hospital indicated that medium to high levels of ER-alpha and PR expression were present in the early stage (FIGO Stages I and II), while low to medium levels of expression were observed in advanced stage patients (FIGO Stages III and IV) (Fig. 6c).

### Association of the prognostic signature and tumor infiltrating immune cells

**Fig. 7:**
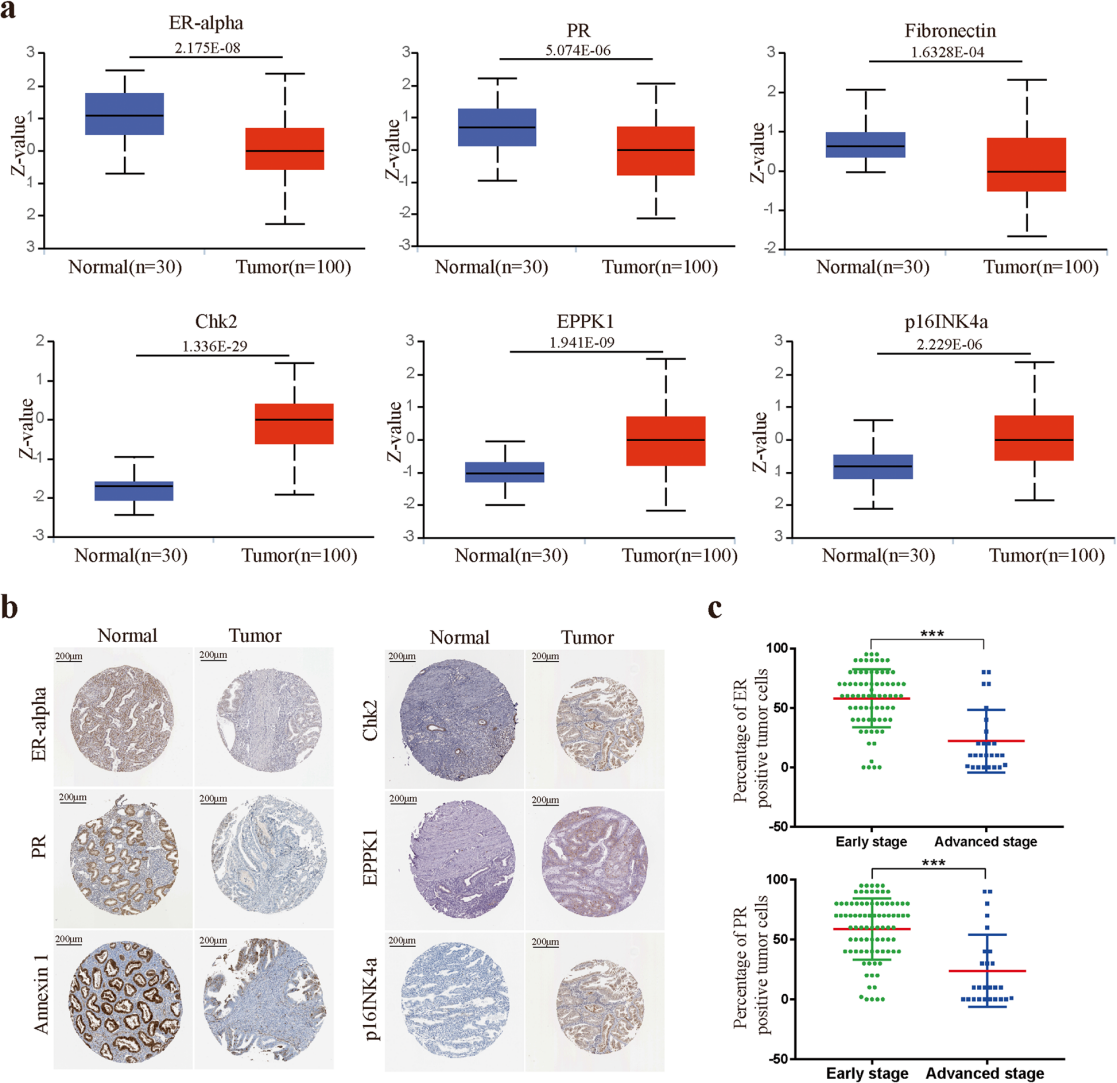
Association of the prognostic signature and tumor infiltrating immune cells. **a** Violin plot comparing the proportions of immune cells between the high-risk and low-risk groups. **b** Survival curves obtained by the Kaplan–Meier method indicated that high proportions of CD8 T cells, T follicular helper cells and regulatory T cells were significantly associated with prolonged OS. **c** Correlation matrix of 22 immune cells and the risk score system

To investigate the correlation of the prognostic signature with tumor infiltrative immune cells in EC samples. The CIBERSORT algorithm was used to quantify the proportions of immune cells in each EC sample. The proportions of CD8 T cells, T follicular helper cells and regulatory T cells were higher in the low-risk group, while M2 macrophages, monocytes and activated dendritic cells showed a higher density in the high-risk group (Fig. 7a). In addition, high infiltration of CD8 T cells, T follicular helper cells and regulatory T cells was correlated with prolonged OS by Kaplan–Meier analysis (Fig. 7b), which was consistent with previous results showing that a low risk score was associated with better prognosis. Correlation analyses of immune cells and the risk score system indicated that the infiltration regulatory T cells were negatively correlated with the risk score system, while macrophage M2 and activated dendritic cells were positively correlated with the risk score system (Fig. 7c). Taken together, these results indicated that the risk score system might negatively reflect the infiltration level of effector T cells in PC tumors, which are responsible for adaptive antitumor immunity.

### TMB, MSI and immunotherapy responsiveness with prognostic signature correlations between the prognostic signature and TMB and MSI status

**Fig. 8:**
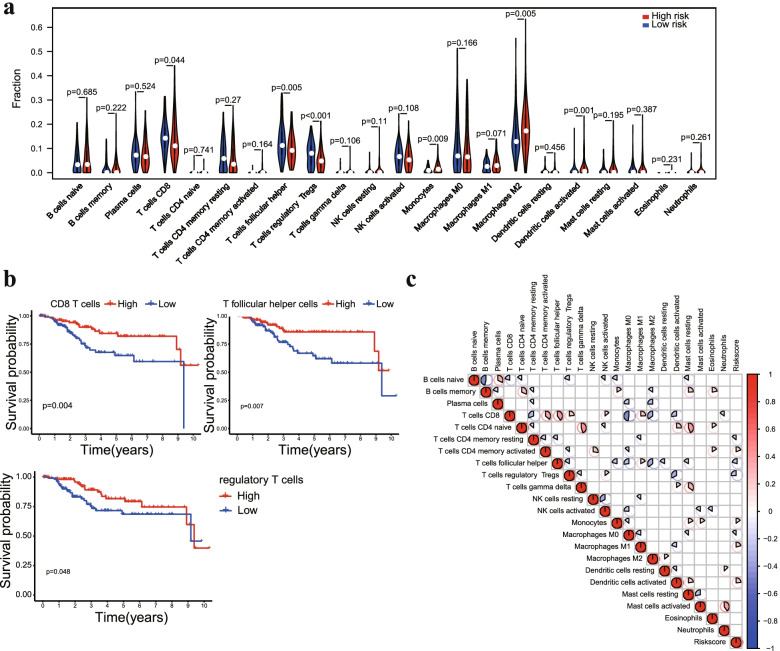
Association of the prognostic signature with TMB and MSI status. **a** Summary of the overall mutation profile of EC patients. **b** The scatter plot shows the correlation between the risk score and TMB value of EC patients. **c** Violin plot of the association of MSI status and risk score. **d** Kaplan–Meier curves showed that MSI-H patients had a favorable prognosis in EC patients

To evaluate the relationship between the prognostic signature and TMB, we analyzed the mutation profile of EC patients. A summary of the overall mutation profile of EC patients is shown in Fig. 8a. The top five mutated genes in EC patients were PTEN, PIK3CA, ARID1A, TP53 and TTN. The mutations were classified according to the variant effect predictor; among these mutations, missense mutations were the most common, and the most common mutation type was SNPs (Fig. S5a). The TMB value in the low-risk group was higher than that in the high-risk group (Fig. 8b). Furthermore, high TMB was associated with more favorable outcomes in EC patients (Fig. S5b). By analyzing the association between the risk score and MSI status, we found that the risk score was significantly decreased in MSI-H patients (Fig. 8c). Patients with MSI-H had a better prognosis than those of patients with MSS or MSI-L (Fig. 8d). Tumor mutational burden (TMB) and MSI status were promising predictive biomarkers for treatment with immune checkpoint inhibitors. These results suggested that the prognostic signature was significantly associated with TMB and MSI status, which has the potential to predict the response to immune checkpoint inhibitors.

### Predicting response to immunotherapy in patients with EC

**Fig. 9:**
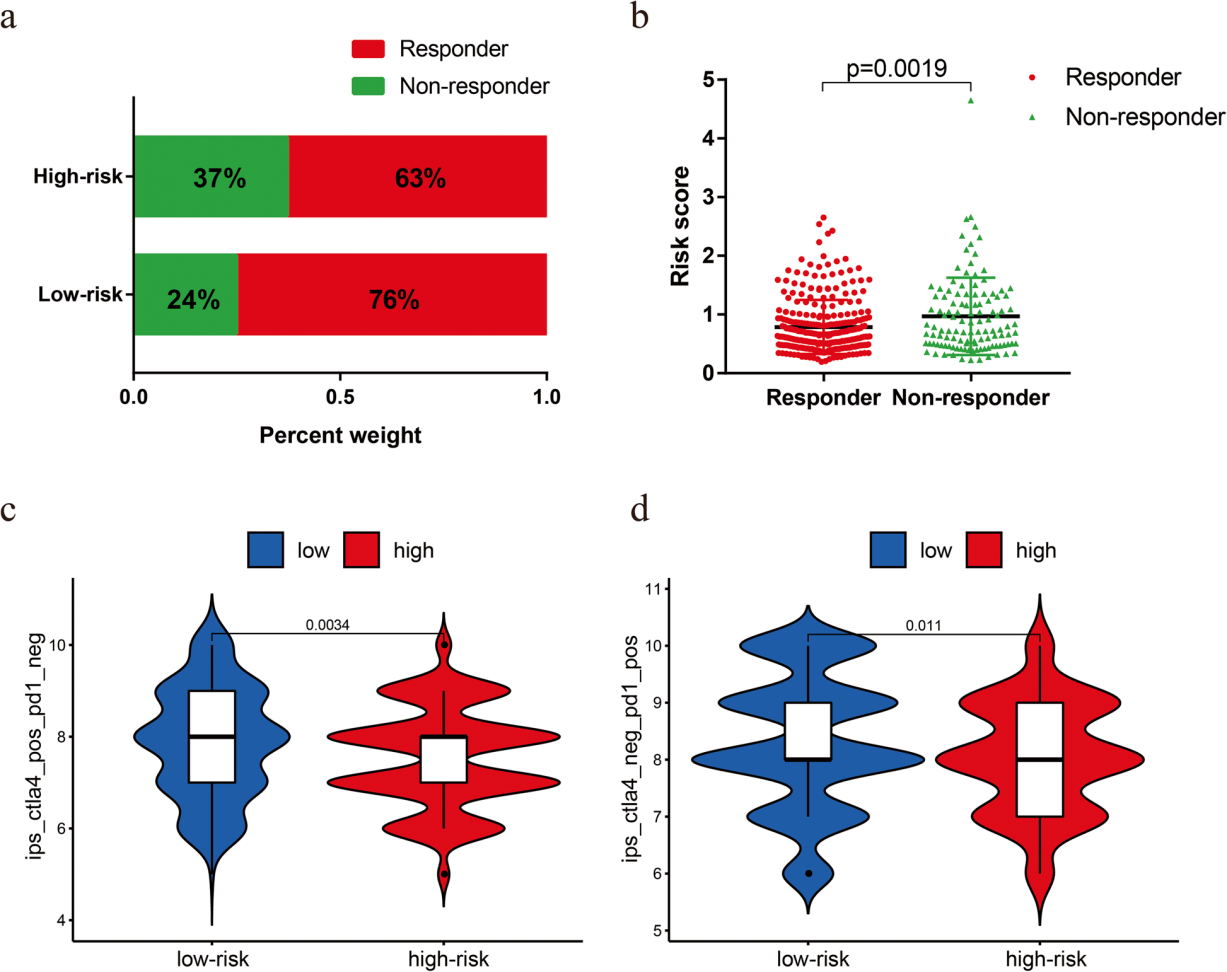
The low-risk group may be more sensitive to immunotherapies. **a** The differences in response results to immunotherapy between low-risk and high-risk groups. **b** The scatter plot shows the correlation between immunotherapy responsiveness and risk score in EC patients. **c** The relative probabilities of responding to anti-CTLA-4 antibody in the low-risk and high-risk groups. **d** The relative probabilities of responding to anti-PD-1/PD-L1 antibody in the low-risk and high-risk groups

Finally, we evaluated the potential immunotherapy response in each patient by the ImmuCellAI algorithm. The results showed that patients in the low-risk group (76%, 153/201) were more likely to respond to immune checkpoint blockade than were patients in the high-risk group (63%, 127/202) (Fig. 9a). In addition, the risk score was lower in the responders than in the nonresponders (Fig. 9b). The Kaplan–Meier curve showed better survival in the responder group than in the nonresponder group (Fig. S5c). In addition, we further applied TCIA to predict the susceptibility of patients to immunotherapy. We found that the low-risk group had a higher IPS than that of the high-risk group, which meant that the low-risk group may be more sensitive to immune checkpoint inhibitors (Fig. 9c-d). Taken together, these results indicated that the prognostic signature could predict the potential response to immunotherapy in EC patients.

**Fig. 10:**
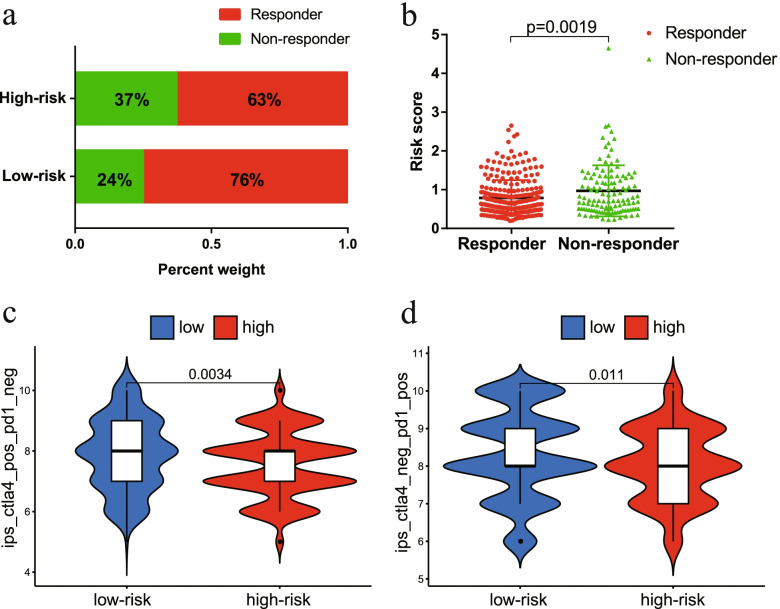
**a** Evaluation of the sensitivity of chemotherapy drugs between the high-risk and low-risk groups based on the IC50 values of paclitaxel, cisplatin and doxorubicin for EC patients. **b** Differences in molecular drug sensitivity between the high-risk and low-risk groups based on IC50 values of AKT inhibitor VIII, VEGFR inhibitor (pazopanib) and mTOR inhibitor (temsirolimus)

In addition, we also evaluated the relationship between the model and the sensitivity to chemotherapy and targeted therapy for EC patients. Our results showed that the IC50 values of paclitaxel, cisplatin and doxorubicin were significantly higher in samples of the low-risk group than in those of the high-risk group (Fig. 10a). Interestingly, the high-risk group demonstrated much higher sensitivity to the AKT inhibitor VIII, VEGFR inhibitor (pazopanib) and mTOR inhibitor (temsirolimus) than that of the low-risk group (Fig. 10b). These results indicated that the risk score had potential predictive value for chemotherapy and targeted therapy.

## Discussion

At present, almost all risk stratification systems in EC use a composite of histology, stage and grade. Recent studies have shown that cancers of the same stage and histology have distinctive molecular and genomic profiles [8]. Proteogenomic expression data have enabled a comprehensive understanding of the prognosis of cancer. Although the application of next-generation sequencing (NGS) makes the analysis of RNA and DNA levels popular in clinical oncology, it is worth noting that most genes need to play a role at the protein level. Systematic investigation of protein expression profiling provided an essential approach to uncover interactions between the immune system and tumor components in patients, providing useful information for cancer prognosis or guiding treatment decisions. The proteomics signature has been widely studied in various tumors, including renal cell carcinoma, head and neck squamous cell carcinoma, colorectal cancer, hepatocellular carcinoma, lung squamous cell carcinoma and bladder urothelial carcinoma. However, to date, there are relatively few studies exploiting the role of proteomic models in EC patients.

In this study, we acquired 45 prognostic proteins based on univariate regression analysis. Then, we used the LASSO-Cox algorithm to develop a prognostic signature composed of 9 proteins and calculated the risk score for each patient. Patients were divided into high-risk and low-risk groups by the risk scores of the prognostic signature. EC patients in the low-risk group showed a longer OS than those in the high-risk group in both the training and testing sets. The accuracy of the signature prediction for 1, 3, and 5 years of the three sets was more than 0.7. These results proved its accurate prediction ability and indicated that the signature was a potential prognostic tool for EC patients. Compared with a single biomarker, the combination of multiple proteins showed better predictive efficacy. In addition, the prognostic nomogram consisting of a 9-protein signature and clinicopathological factors may enable medical practitioners to determine individual patient prognosis. Our results also showed that the performance of the protein signature in predicting the prognosis of EC patients was better than the previously published signatures. Furthermore, we also compare proteins identified in our study with previous studies. There was no overlap proteins among these studies. These prognostic proteins or genes were enriched in several molecular pathways. The prognostic signature based on transcriptome dataset may lead some bias as our signature derived from proteomic data.

Among these 9 proteins in our prognostic signature, 4 proteins (Chk2-pT68, Fibronectin, EPPK1, and p16INK4a) were associated with risk factors, and 5 proteins (X1433EPSILON, ER-alpha, PR, Annexin 1, and Myosin IIA) were related to protective factors. Our study found that high expression levels of Chk2-pT68 and p16INK4a were related to higher tumor grade, clinical stage and microsatellite stability, which were associated with a lower OS rate of EC patients. Chk2 is an enzyme that is a key component of the DNA damage response. These data were in conflict with other studies where Chk2 protein was shown to be a tumor suppressor and to act as a good prognostic indicator in other tumor types [27, 28]. These distinct associations with patient prognosis may be explained by the complex function of Chk2 and its complicated interactions with other key cancer proteins. p16INK4a is a tumor suppressor protein that has a crucial role in the process of cellular senescence in progenitor cells [29]. The increased expression of p16INK4a in the high-risk group in this study indicated the importance of cellular senescence in tumor progression. Correspondingly, we also observed that higher expression levels of ER-alpha and Annexin 1 were related to lower tumor grade, clinical stage and microsatellite stability. ER-alpha is a member of the steroid/nuclear receptor superfamily and functions as a signal transducer and transcription factor to regulate target gene expression. Studies with EC and breast cancers have shown that a high expression level of ER-alpha was associated with a favorable prognosis and a good response to treatment [30, 31]. These findings were consistent with our results that EC patients with high levels of ER-alpha had a longer OS. Furthermore, IHC staining in EC tissues of our hospital demonstated that high expression of ER-alpha and PR were positively correlated with FIGO Stages. Compared to those of EC from patients with advanced stage, ER-alpha and PR expression in EC from patients with early stage were significantly increased. Annexin 1 belongs to the annexin family, which plays important roles in inflammatory modulation and the immune response [32]. The loss of Annexin 1 expression in esophageal cancer, prostate cancer and breast cancer was correlated with metastasis and poor prognosis [33, 34]. Therefore, our results may suggest that Annexin 1 may serve as a negative biomarker in cancer development and in the progression of EC.

Studies based on immune infiltrating cell composition, immune microenvironment and immunotherapeutic targets have been applied and conducted in clinical trials [35–37]. To obtain better insights into the functional roles of the immune cell infiltration of EC, the relationships between the protein-based prognostic signature and immune cell infiltration were investigated. Our results showed that CD8 T cells, T follicular helper cells and regulatory T cells were higher in the low-risk group, which indicated that the high infiltration of immune cells may contribute to a good prognosis. The microenvironment of EC cells settles in diverse cell types, including endothelial cells, fibroblasts, myofibroblasts, and immune and inflammatory components. Dynamic reciprocity between tumor cells and immune cells plays a crucial role in tissue homeostasis and tumor growth [38]. Several studies have demonstrated that tumor-infiltrating immune cells are associated with prognosis in EC [39, 40]. Our results proved the upregulation of antitumor immune activity in the low-risk group, which partially explained the predictive value of the prognostic signature.

Cancer immunotherapy, which works by activating the systemic immune response or restoring tumor-induced immune deficiencies, has been successful in treating a variety of malignancies [41]. Immune checkpoint inhibitors targeting the PD-1/PD-L1 pathway were reported to be effective in advanced or metastatic EC patients in several case reports [42, 43]. A recent clinical trial investigating the effect of pembrolizumab in advanced EC patients showed that partial response was achieved in 3 patients [44]. However, only a minority of patients benefited from immunotherapy. Findings in selected cancer types, including EC, suggested that TMB may predict the clinical response to immune checkpoint inhibitors [45, 46]. Budczies et al. reported that high TMB was positively correlated with MSI-H status and related to good survival in EC patients [47]. Consistent with these results, in this study, we found that the low-risk group had a higher TMB value, and EC patients with a high TMB had a significantly better prognosis. Furthermore, we found that MSI-H was negatively correlated with the risk score and improved survival outcome in EC patients. These results might partly explain why patients in the low-risk group possessed better survival than those in the high-risk group. In addition, our results implied that the risk score model may be a good predictor of immunotherapy. Cytotoxic chemotherapeutic agents including paclitaxel, doxorubicin and cisplatin were first line drugs for EC patients. In present study, we found that high-risk group exhibited more sensitive to chemotherapy in comparison to low-risk group by using the GDSC database. Recently, targeted therapies including PI3K/Akt/mTOR inhibitors and VEGF inhibitor have been confirmed to play an important role in the treatment of EC patients. Interestingly, our results also demonstrated that the low-risk group was more sensitive to the AKT inhibitor, mTOR inhibitor and VEGF inhibitor. These results further support the potential of the prognostic protein signature to predict treatment sensitivity for EC patients.

However, there were a few limitations in our study. First, this signature was developed based on publicly available datasets. The lack of external independent validation resulted in limited clinical value of our model. Future work needs to validate the signature using clinical samples of our hospital. Second, molecular biology experiments should be carried out to explore potential functions of these proteins and clarify the underlying molecular mechanism of the proteomic signature.

## Conclusions

In summary, we constructed a valid prognostic protein signature to predict the prognosis of EC patients. The prognostic signature was closely associated with high TMB and MSI-H status and provided potential therapeutic targets for the improvement in treatment in EC patients. We also demonstrated that the prognostic signature had reliable potential to predict the response to immunotherapy, chemotherapy and targeted therapy.

## Supplementary Information

**Additional file1:** **Table S1.** Clinicopathological characteristics of EC patients in the training and testing sets. **Fig. S1** a LASSO regression analysis of 45 prognostic proteins. b Calculation of the C-index of each the prognostic signature. **Fig. S2** a Univariate and multivariate Cox regression analysis demonstrates that the prognostic signature was an independent prognostic factor in the testing set. b Univariate and multivariate Cox regression analysis demonstrates that the prognostic signature was an independent prognostic factor in the total set. **Fig. S3** Differences between our signature and other signatures of previously published studies in EC. a Identification of independent prognostic factors and establishment of the nomogram. b C-index value of our protein signature and the other four risk signatures. c Comparison of 1-year, 3-year, and 5-year ROC curves for our signature with four other risk signatures. **Fig. S4** The expression of the 9 proteins was related to tumor grade a and MSI status b in EC patients. **Fig. S5** a Variant classification and type of genetic alterations in EC. b Kaplan-Meier curves showed that high TMB patients had favorable prognosis in EC patients. c Kaplan-Meier curves showed that responders had favorable prognosis.

## Data Availability

The datasets analyzed for this study can be found in the TCPA (https://www.tcpaportal.org/tcpa/) and TCGA (https://portal.gdc.cancer.gov/).

## Abbreviations

EC: Endometrial cancer
TCPA: The Cancer Proteome Atlas
TCGA: The Cancer Genome Atlas
LASSO: The least absolute shrinkage and selection operator
TCIA: The Cancer Immunome Atlas
TMB: Tumor mutation burden
MSI: Microsatellite instability
ROC: Receiver operating characteristic
AUC: Area under the curve
OS: Overall survival
HR: Hazard ratio
PCA: Principal component analysis
IPS: Immunophenscore
NGS: Next-generation sequencing
FFPE: Formalin-fixed paraffin-embedded
IHC: Immunohistochemistry
CPTAC: Clinical Proteomic Tumor Analysis Consortium
HPA: Human Protein Atlas
