# Correction: Protein-based prognostic signature for predicting the survival and immunotherapeutic efficiency of endometrial carcinoma

**DOI:** 10.1186/s12885-022-09517-0

**Published:** 2022-05-02

**Authors:** Jinzhi Lai, Tianwen Xu, Hainan Yang

**Affiliations:** 1 Department of Oncology, The Second Affiliated Hospital of Fujian Medical University, Quanzhou, 362000 Fujian China; 2 Department of Ultrasound, First Affiliated Hospital of Xiamen University, Xiamen, 361000 Fujian China

**Correction: BMC Cancer 22, 325 (2022)**

**https://doi.org/10.1186/s12885-022-09402-w**

Following publication of the original article [1], an error was identified regarding the order of Figs. 5, 6, 7, 8, 9 and 10.

- Fig. S3 was incorrectly published as Fig. 5.
- Fig. 5 was incorrectly published as Fig. 6.
- Fig. 6 was incorrectly published as Fig. 7.
- Fig. 7 was incorrectly published as Fig. 8.
- Fig. 8 was incorrectly published as Fig. 9.
- Fig. 9 was incorrectly published as Fig. 10.
- We missed to upload the corrected version of Fig. 10 when we proofed this article. Fig. 10 has been corrected to maintain the integrity of our article.

**Fig. 5:**
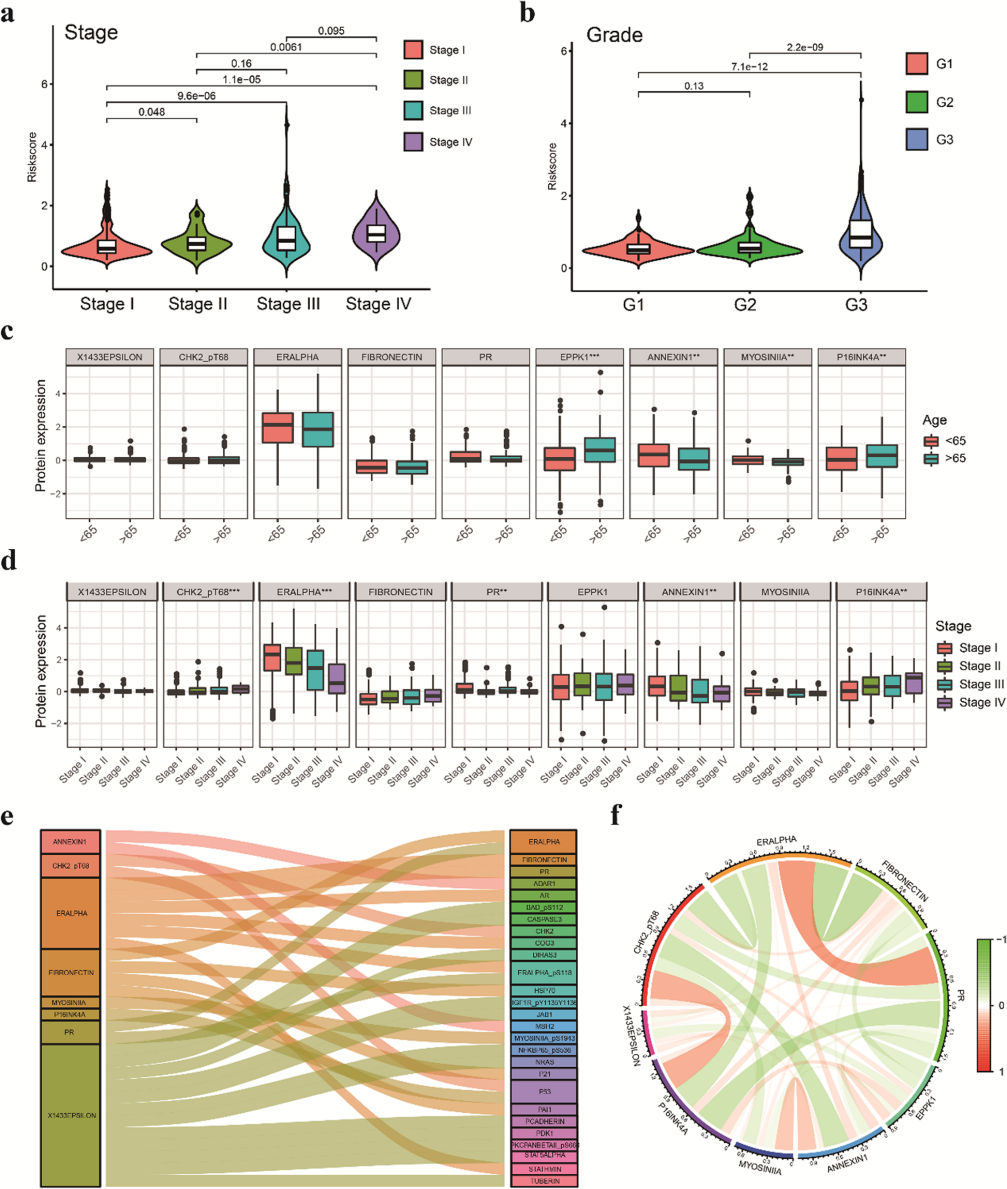
The relationship between 9 prognostic proteins, the risk score and clinical characteristics. **a** The scatter plot shows the correlation between the risk score and tumor stages. **b** The scatter plot shows the correlation between risk score and tumor grade. **c** The expression of EPPK1, p16INK4a, Annexin 1 and Myosin IIA was related to age in EC patients. **d** The expression of ER-alpha, Annexin 1, Chk2-pT68 and p16INK4a was significantly associated with cancer stage. **e** Sankey diagram of all proteins related to 9 proteins in the TCPA database (correlation coefficient > 0.4) (*p* < 0.001). (F) The corelationship of 9 proteins in the prognostic signature. * *p* < 0.05, ** *p* < 0.01, *** *p* < 0.001

**Fig. 6:**
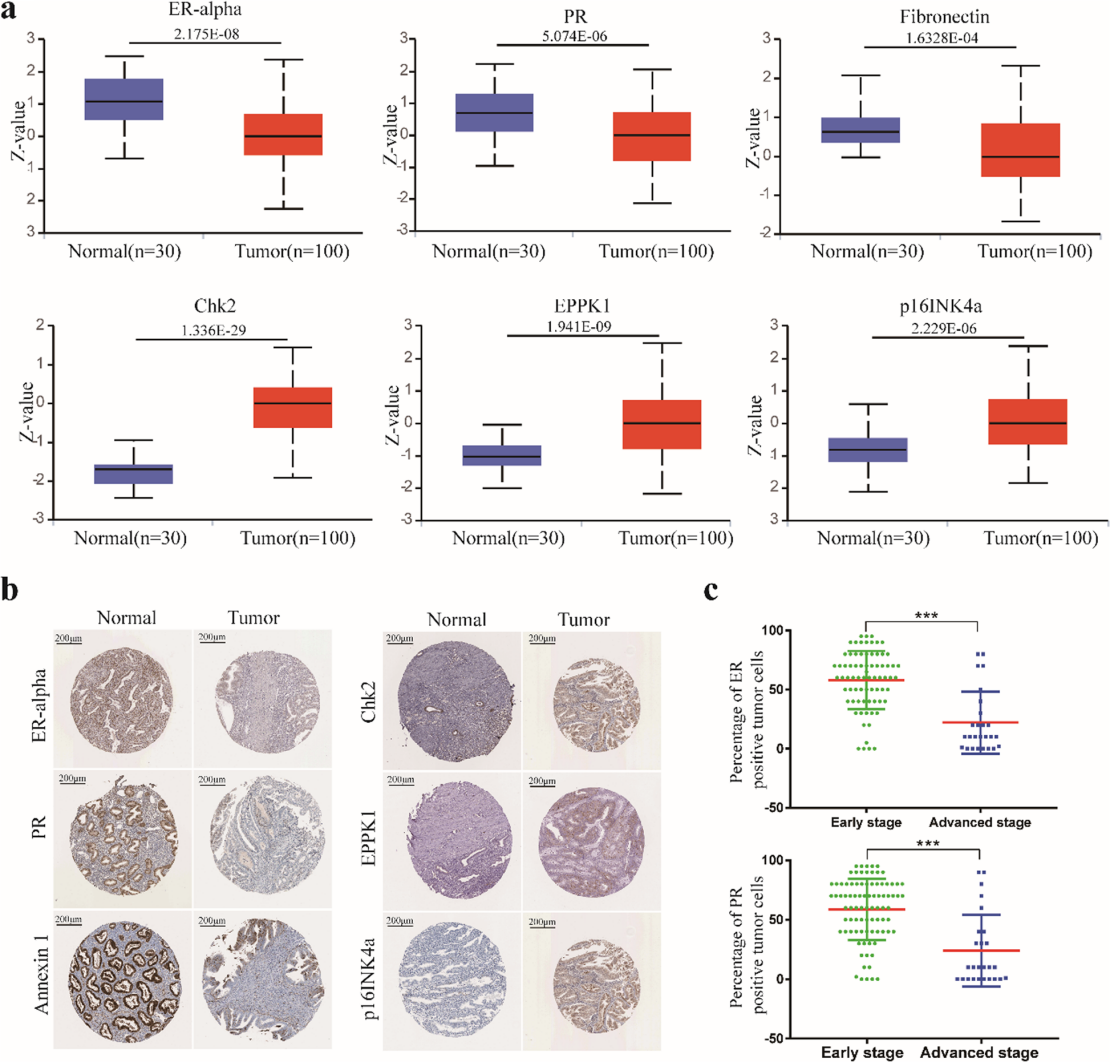
The relationship between 9 prognostic proteins, the risk score and clinical characteristics. **a** Protein level of prognostic proteins in EC tumor tissues and normal tissues. **b** Representative protein expression levels of ER-alpha, PR, Annexin 1, Chk2-pT68, EPPK1, p16INK4a and ASNS explored in the HPA database. **c** IHC staining data of ER-alpha and PR expression levels from 100 clinical samples in our hospital. * *p* < 0.05, ** *p* < 0.01, *** *p* < 0.001

**Fig. 7:**
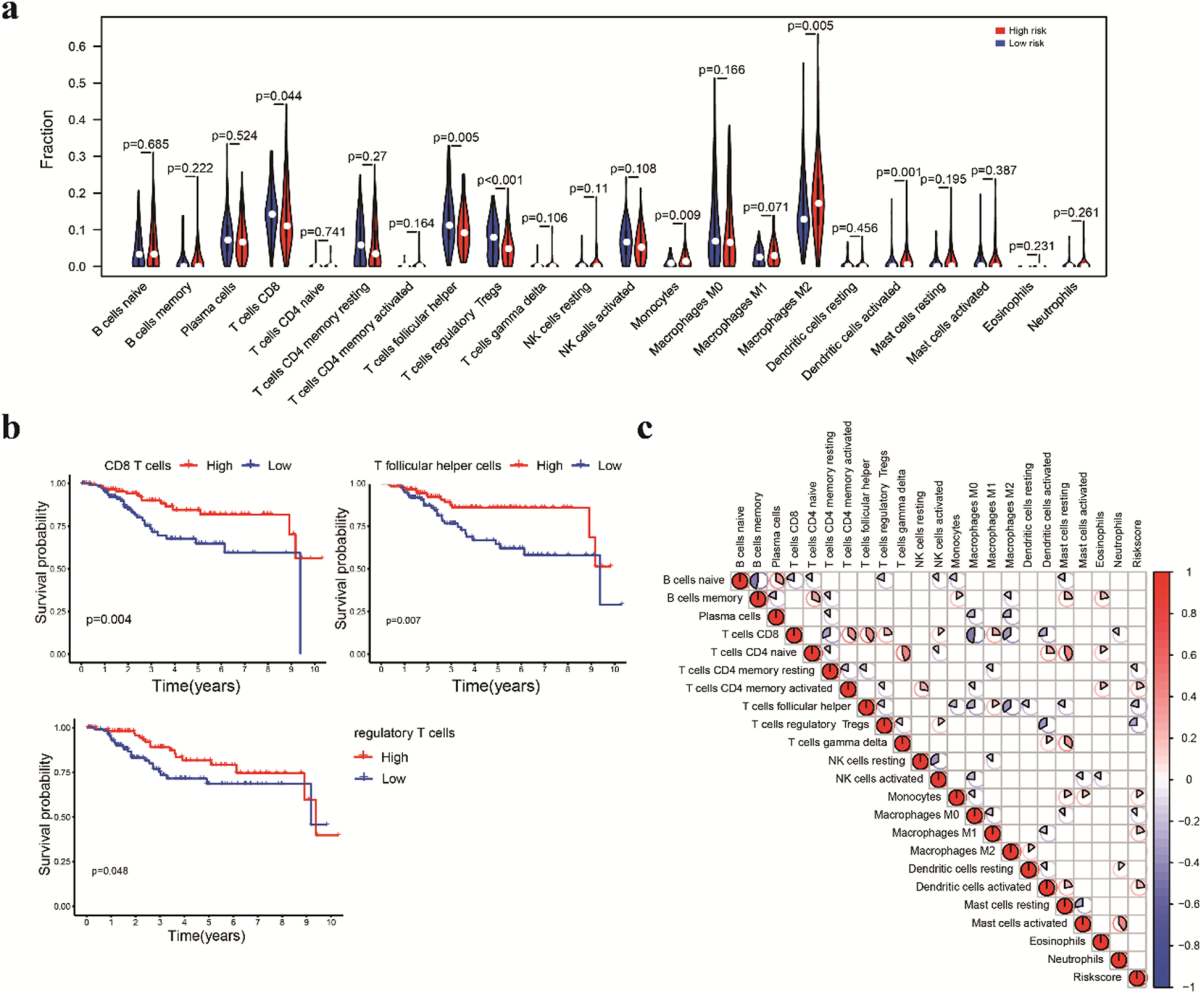
Association of the prognostic signature and tumor infiltrating immune cells. **a** Violin plot comparing the proportions of immune cells between the high-risk and low-risk groups. **b** Survival curves obtained by the Kaplan–Meier method indicated that high proportions of CD8 T cells, T follicular helper cells and regulatory T cells were significantly associated with prolonged OS. **c** Correlation matrix of 22 immune cells and the risk score system

**Fig. 8:**
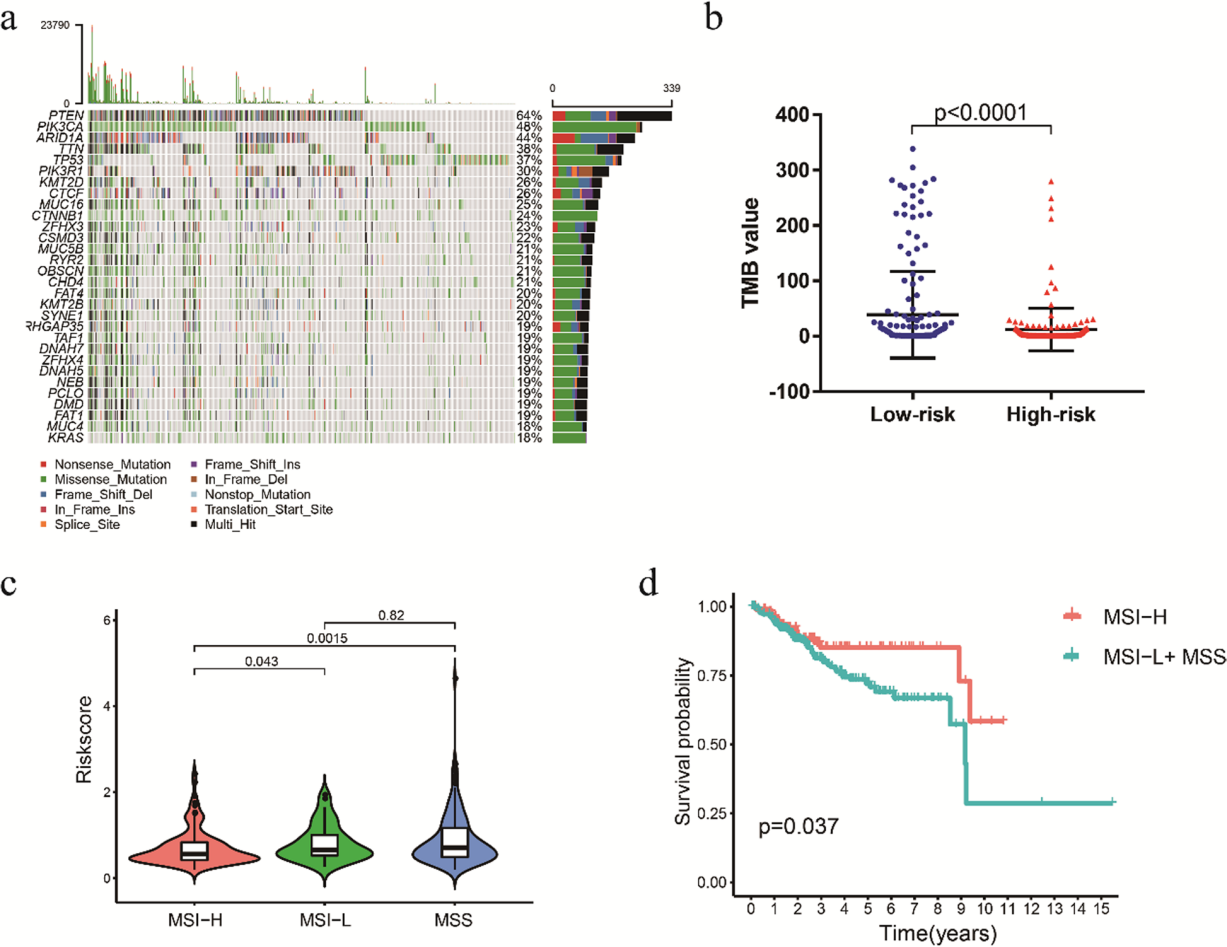
Association of the prognostic signature with TMB and MSI status. **a** Summary of the overall mutation profile of EC patients. **b** The scatter plot shows the correlation between the risk score and TMB value of EC patients. **c** Violin plot of the association of MSI status and risk score. **d** Kaplan–Meier curves showed that MSI-H patients had a favorable prognosis in EC patients

**Fig. 9:**
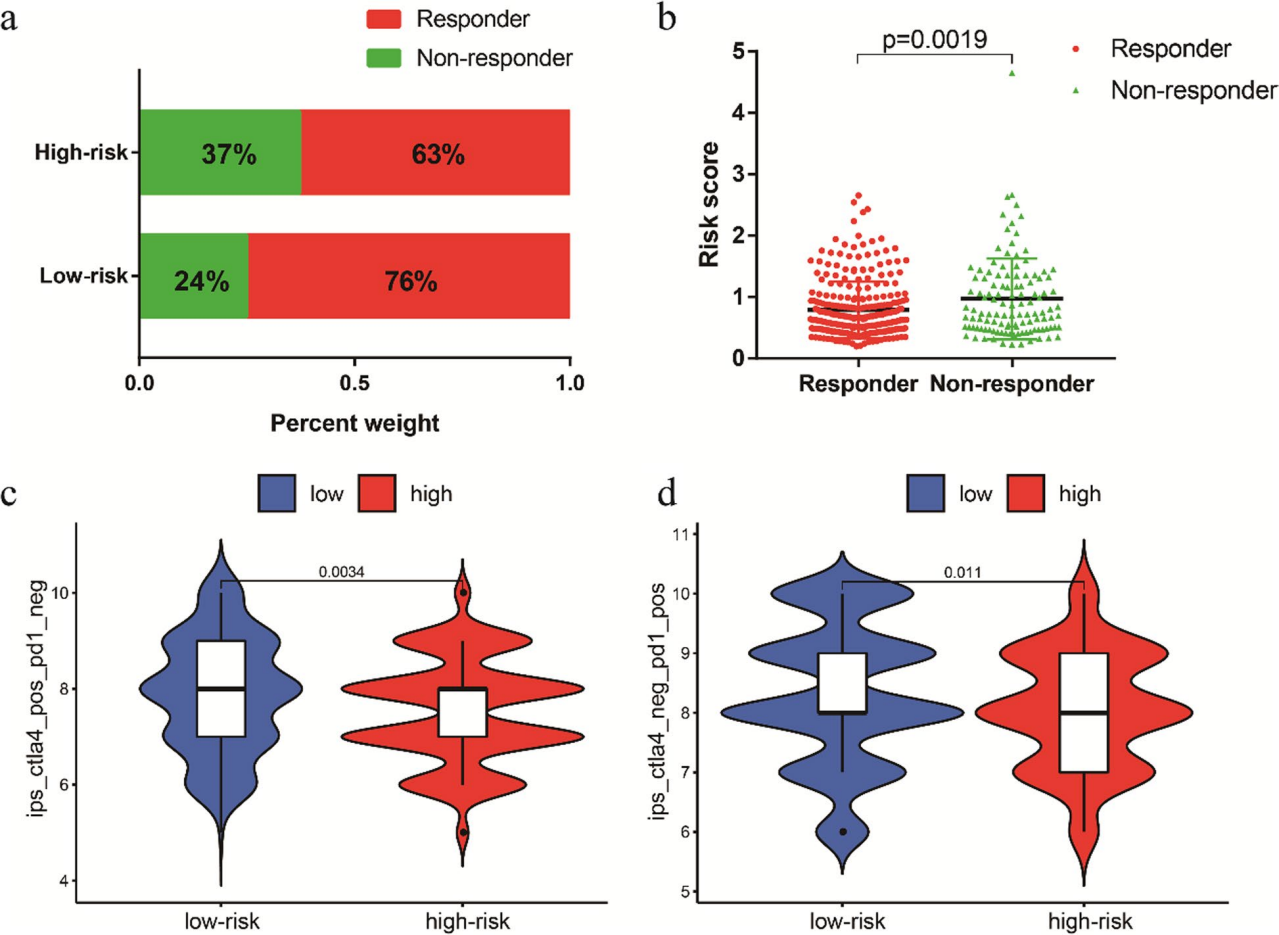
The low-risk group may be more sensitive to immunotherapies. **a** The differences in response results to immunotherapy between low-risk and high-risk groups. **b** The scatter plot shows the correlation between immunotherapy responsiveness and risk score in EC patients. **c** The relative probabilities of responding to anti-CTLA-4 antibody in the low-risk and high-risk groups. **d** The relative probabilities of responding to anti-PD-1/PD-L1 antibody in the low-risk and high-risk groups

**Fig. 10:**
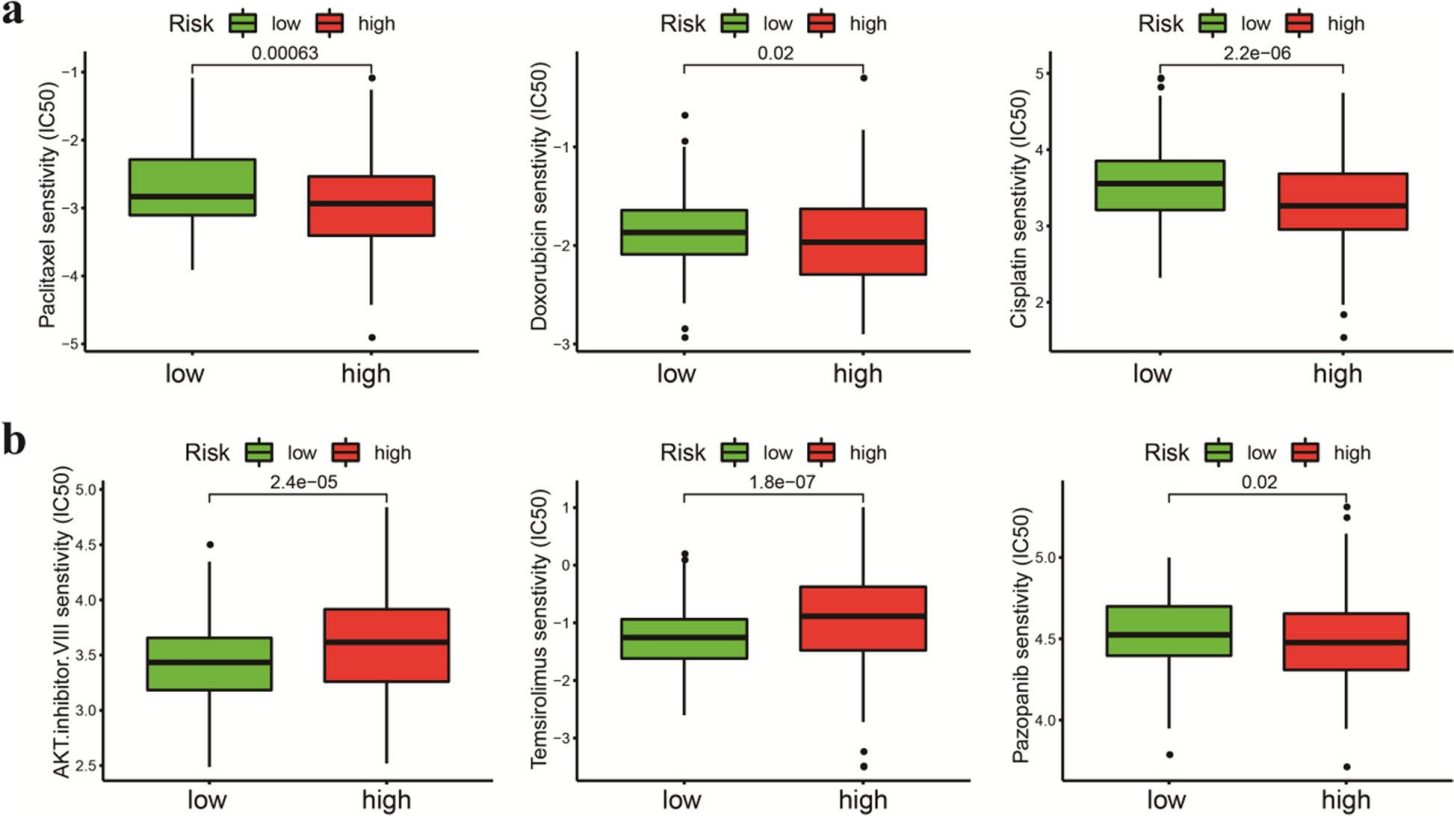
**a** Evaluation of the sensitivity of chemotherapy drugs between the high-risk and low-risk groups based on the IC50 values of paclitaxel, cisplatin and doxorubicin for EC patients. **b** Differences in molecular drug sensitivity between the high-risk and low-risk groups based on IC50 values of AKT inhibitor VIII, VEGFR inhibitor (pazopanib) and mTOR inhibitor (temsirolimus)

The correct versions of Figs. 5, 6, 7, 8, 9 and 10 are given in this correction article. The original article [1] has been corrected.
