# Supplementary material for: Protein-based prognostic signature for predicting the survival and immunotherapeutic efficiency of endometrial carcinoma
Source: BMC Cancer. 2022 Mar 25;22:325. doi: 10.1186/s12885-022-09402-w (PMC8957185; doi:10.1186/s12885-022-09402-w)
Supplement: Supplementary file 1 — Additional file1: Table S1. Clinicopathological characteristics of EC patients in the training and testing sets. Fig. S1 a LASSO regression analysis of 45 prognostic proteins. b Calculation of the C-index of each the prognostic signature. Fig. S2 a Univariate and multivariate Cox regression analysis demonstrates that the prognostic signature was an independent prognostic factor in the testing set. b Univariate and multivariate Cox regression analysis demonstrates that the prognostic signature was an independent prognostic factor in the total set. Fig. S3 Differences between our signature and other signatures of previously published studies in EC. a Identification of independent prognostic factors and establishment of the nomogram. b C-index value of our protein signature and the other four risk signatures. c Comparison of 1-year, 3-year, and 5-year ROC curves for our signature with four other risk signatures. Fig. S4 The expression of the 9 proteins was related to tumor grade a and MSI status b in EC patients. Fig. S5 a Variant classification and type of genetic alterations in EC. b Kaplan-Meier curves showed that high TMB patients had favorable prognosis in EC patients. c Kaplan-Meier curves showed that responders had favorable prognosis. [file 12885_2022_9402_MOESM1_ESM.docx]

Supplementary Material

## Supplementary Table

Table.S1 Clinicopathological characteristics of EC patients in the training and testing sets.

| Characteristics | Training set  (n = 200) | | Testing set  (n = 199) | |
| --- | --- | --- | --- | --- |
|  | Number | % | Number | % |
| **Age (years)** | | | | |
| ≥ 65 | 107 | 53.5 | 102 | 51.3 |
| < 65 | 93 | 46.5 | 97 | 48.7 |
| **Stage** | | | | |
| Stage І | 120 | 60.0 | 125 | 62.9 |
| Stage Ⅱ | 21 | 10.5 | 17 | 8.50 |
| Stage Ⅲ | 47 | 23.5 | 44 | 22.1 |
| Stage Ⅳ | 12 | 6.00 | 13 | 65.5 |
| **Grade** | | | | |
| G1 | 42 | 21.0 | 32 | 16.1 |
| G2 | 39 | 19.5 | 52 | 26.1 |
| G3 | 119 | 59.5 | 115 | 57.8 |
| **MSI** | | | | |
| MSI-H | 60 | 30.0 | 68 | 34.1 |
| MSI-L | 19 | 9.50 | 20 | 10.1 |
| MSS | 121 | 60.5 | 111 | 55.8 |

**Supplementary Figures**


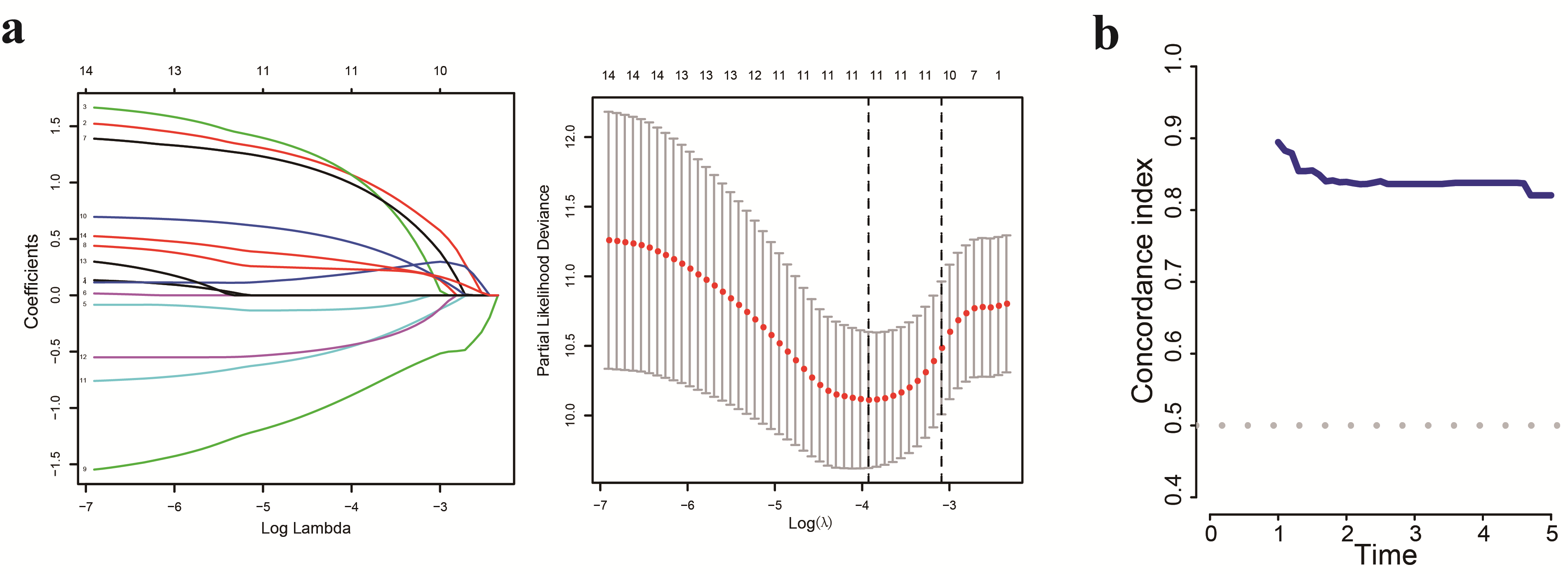


**Fig. S1** **a** LASSO regression analysis of 45 prognostic proteins. **b** Calculation of the C-index of each the prognostic signature.


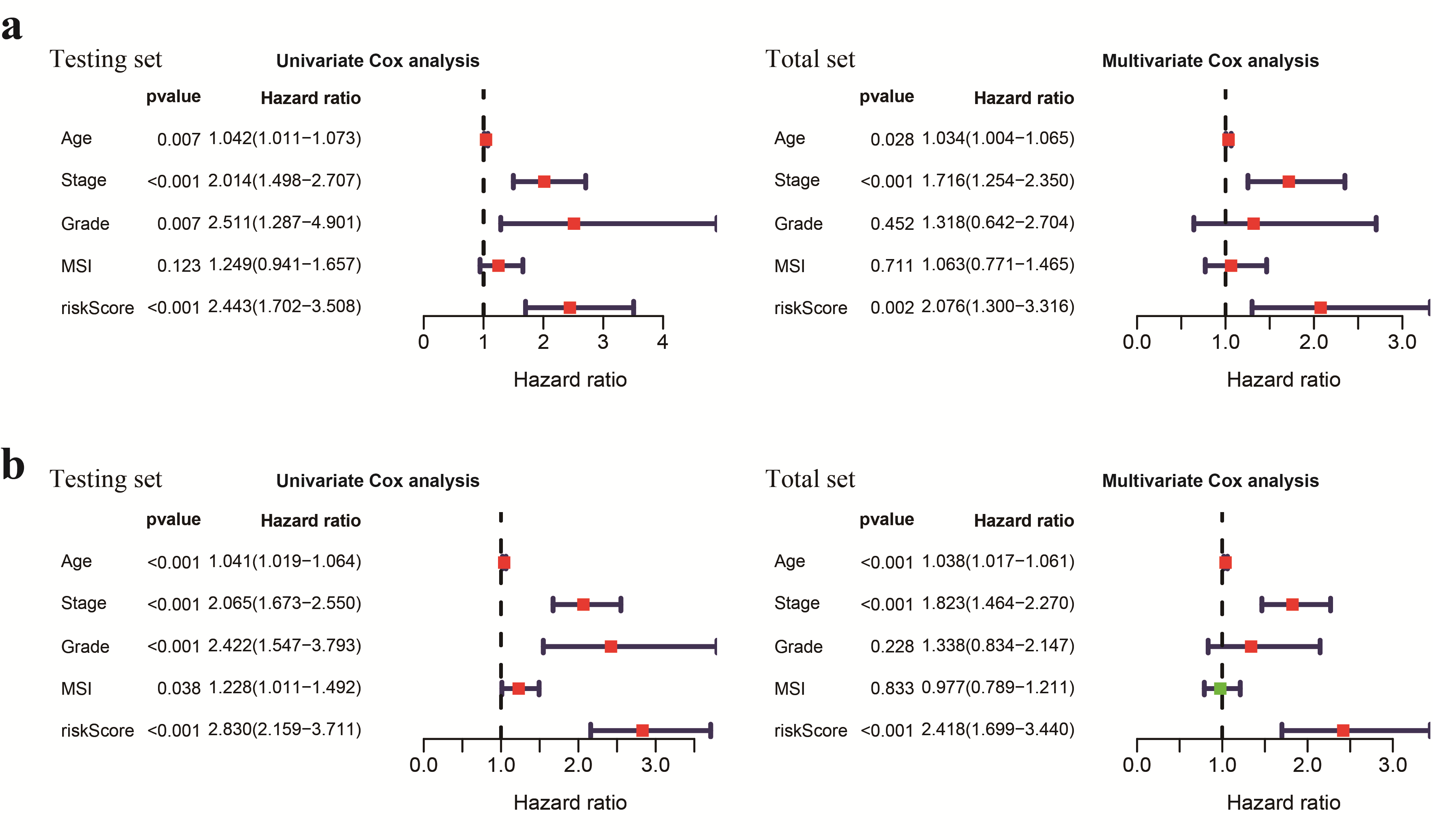


**Fig. S2** **a** Univariate and multivariate Cox regression analysis demonstrates that the prognostic signature was an independent prognostic factor in the testing set. **b** Univariate and multivariate Cox regression analysis demonstrates that the prognostic signature was an independent prognostic factor in the total set.


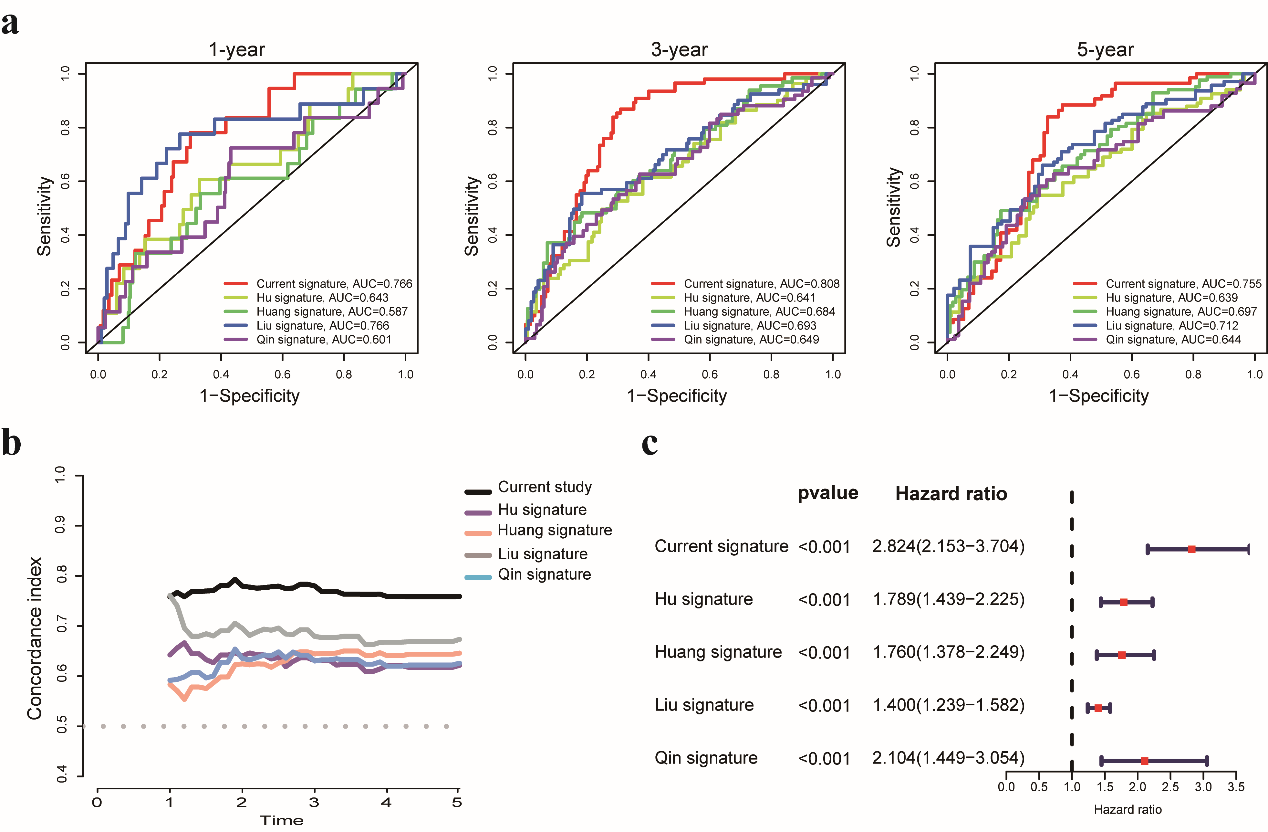


**Fig. S3** Differences between our signature and other signatures of previously published studies in EC. **a** Identification of independent prognostic factors and establishment of the nomogram. **b** C-index value of our protein signature and the other four risk signatures. **c** Comparison of 1-year, 3-year, and 5-year ROC curves for our signature with four other risk signatures.


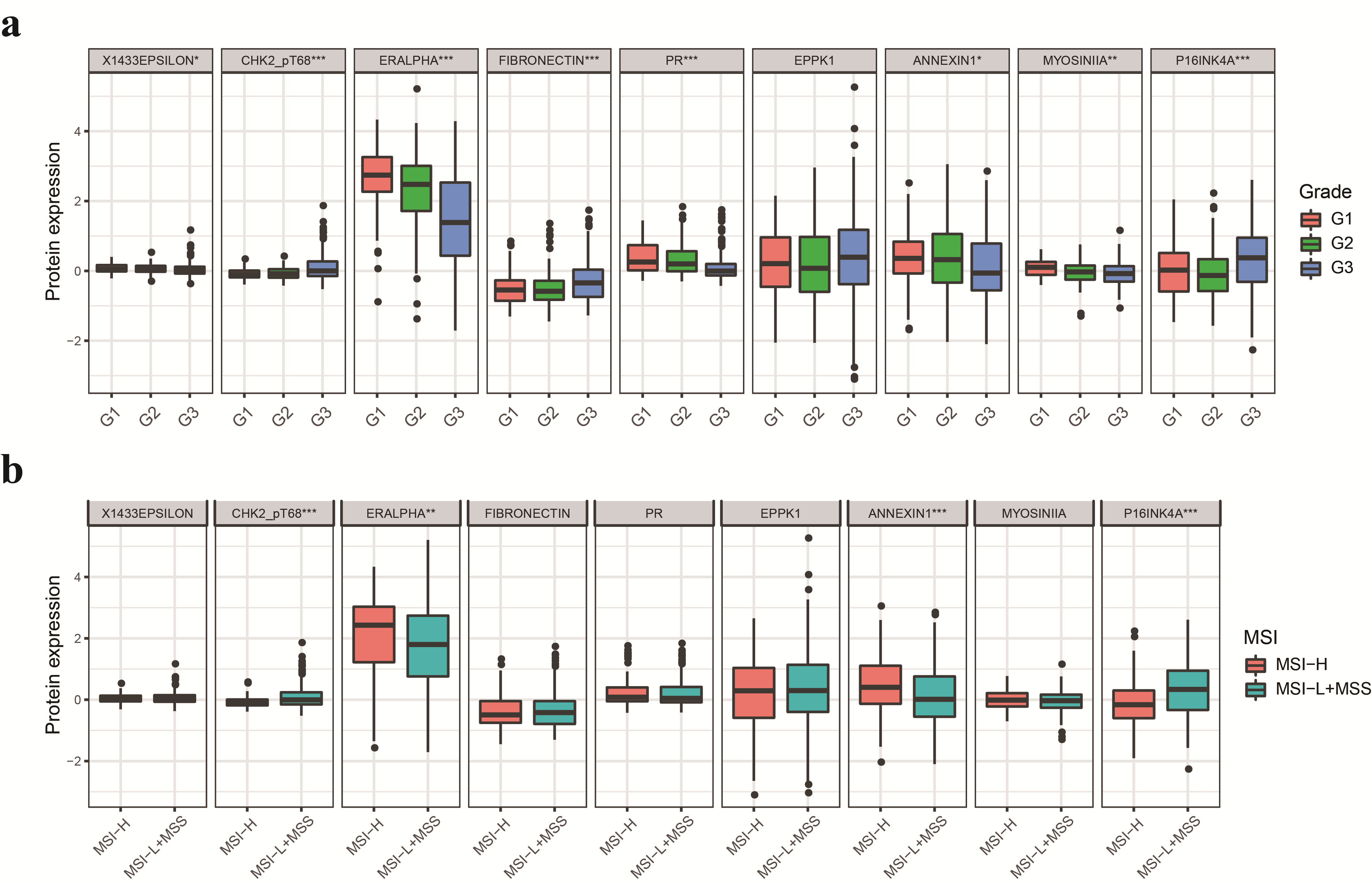


**Fig. S4**: The expression of the 9 proteins was related to tumor grade **a** and MSI status **b** in EC patients.


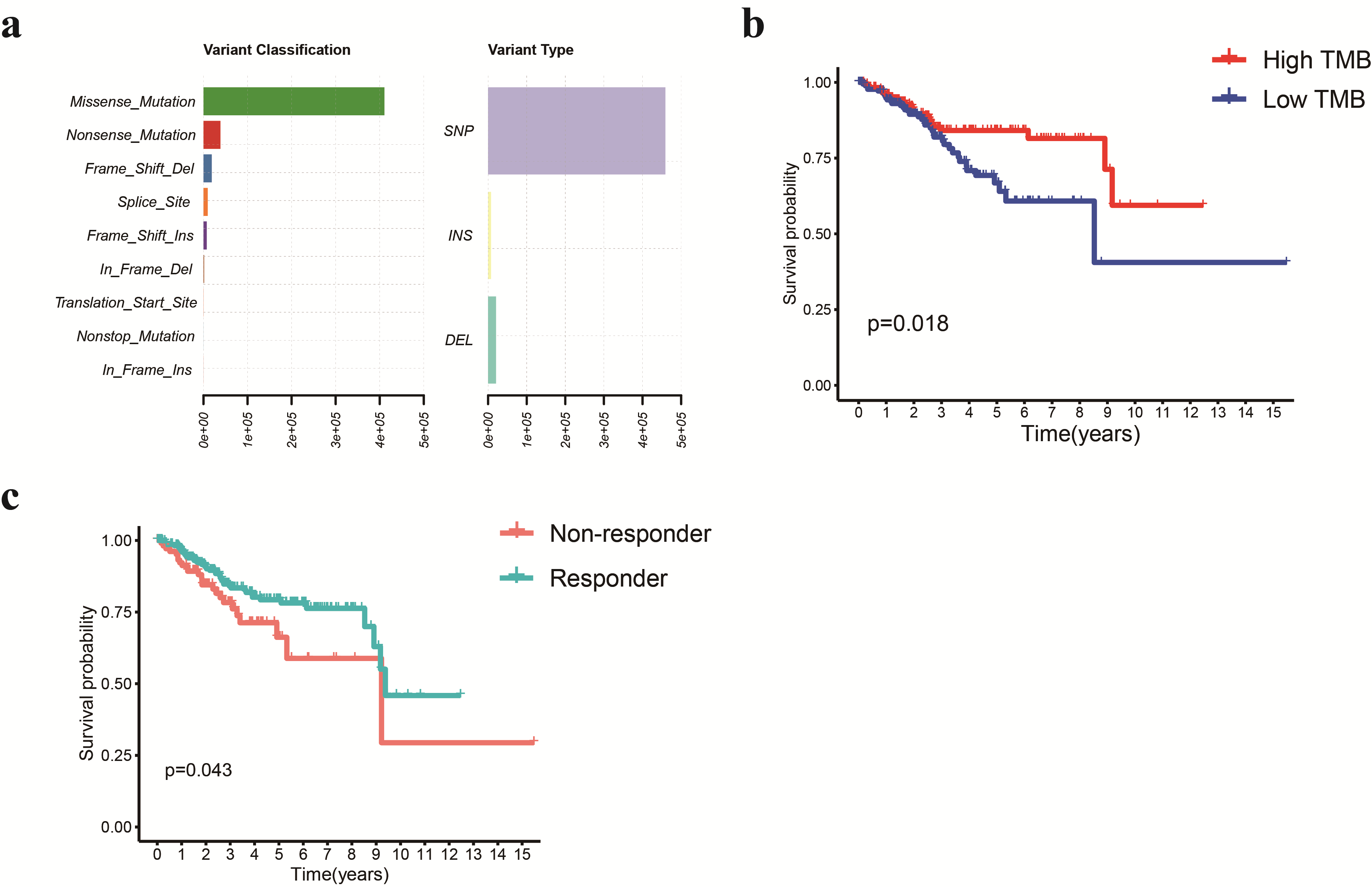


**Fig. S5**: **a** Variant classification and type of genetic alterations in EC. **b** Kaplan-Meier curves showed that high TMB patients had favorable prognosis in EC patients. **c** Kaplan-Meier curves showed that responders had favorable prognosis.
